# Nitrogen Deposition Shifts Grassland Communities Through Directly Increasing Dominance of Graminoids: A 3-Year Case Study From the Qinghai-Tibetan Plateau

**DOI:** 10.3389/fpls.2022.811970

**Published:** 2022-03-04

**Authors:** Hao Shen, Shikui Dong, Antonio DiTommaso, Jiannan Xiao, Wen Lu, Yangliu Zhi

**Affiliations:** ^1^School of Grassland Science, Beijing Forestry University, Beijing, China; ^2^State Key Joint Laboratory of Environmental Simulation and Pollution Control, School of Environment, Beijing Normal University, Beijing, China; ^3^Soil and Crop Sciences, School of Integrative Plant Science, Cornell University, Ithaca, NY, United States; ^4^Department of Natural Resources, Cornell University, Ithaca, NY, United States; ^5^Ministry of Education Key Laboratory of Ecology and Resource Use of the Mongolia Plateau, Collaborative Innovation Center for Grassland Ecological Security, College of Ecology and Environment, Inner Mongolia University, Hohhot, China

**Keywords:** alpine grassland, community productivity, N deposition, species composition, species richness, Qinghai-Tibetan Plateau

## Abstract

Nitrogen (N) deposition has been increasing for decades and has profoundly influenced the structure and function of grassland ecosystems in many regions of the world. However, the impact of N deposition on alpine grasslands is less well documented. We conducted a 3-year field experiment to determine the effects of N deposition on plant species richness, composition, and community productivity in an alpine meadow of the Qinghai-Tibetan Plateau of China. We found that 3 years of N deposition had a profound effect on these plant community parameters. Increasing N rates increased the dominance of graminoids and reduced the presence of non-graminoids. Species richness was inversely associated with aboveground biomass. The shift in plant species and functional group composition was largely responsible for the increase in productivity associated with N deposition. Climatic factors also interacted with N addition to influence productivity. Our findings suggest that short-term N deposition could increase the productivity of alpine meadows through shifts in composition toward a graminoid-dominated community. Longer-term studies are needed to determine if shifts in composition and increased productivity will be maintained. Future work must also evaluate whether decreasing plant diversity will impair the long-term stability and function of sensitive alpine grasslands.

## Introduction

Atmospheric nitrogen (N) deposition has been increasing globally for decades (Phoenix et al., 2006; Galloway et al., 2008a). N deposition rates in terrestrial ecosystems are projected to increase to 200 Tg N yr^–1^ by 2050 (Galloway et al., 2008b). As N is the principal limiting nutrient for plant growth (Vitousek and Howarth, 1991), N deposition may have positive effects on plant productivity. These effects may be strongest in N-limited ecosystems (Vitousek et al., 1997) such as alpine grasslands, which are more sensitive to shifts in nitrogen availability (Xu et al., 2018). There is evidence that long-term N deposition can increase the productivity of grassland ecosystems (Bai et al., 2008; Xia and Wan, 2008; Shen et al., 2019), possibly impacting ecosystem function (Gruber and Galloway, 2008; Stevens et al., 2015). High levels of N deposition (72 kg N ha^–1^ year^–1^) in grassland ecosystems have also been shown to alter plant community structure, decreasing plant species richness and altering species composition (Mountford et al., 1993; Bobbink et al., 2010; Pierik et al., 2011; Isbell et al., 2013). As N deposition rates increase, nitrophilous species become more abundant while N-sensitive species become less abundant (Stevens et al., 2004).

Grasslands are important terrestrial ecosystems covering approximately 25% of earth’s land surface (Hui and Jackson, 2006). Species richness and composition are two important components of plant diversity in grassland ecosystems (Marini et al., 2007). The effects of N inputs on species richness and composition may be mediated by changes to multiple soil properties (Myklestad, 2004). Different plant functional groups in a grassland ecosystem may respond differently to N deposition because of contrasting N-use efficiencies and adaptation mechanisms (Harpole and Tilman, 2007; Song et al., 2011). Some grassland studies have highlighted the importance of species richness to ecosystem function by demonstrating that species richness is positively correlated with productivity (Tilman et al., 1996; Hector et al., 1999). However, other research has indicated that plant composition represents a stronger influence on productivity than species richness (Hooper and Vitousek, 1997). Therefore, additional research is needed to assess the importance of species richness and composition in determining grassland productivity under N deposition, especially in N-limited habitats such as alpine meadows.

The Qinghai-Tibetan Plateau (QTP), known as the “third pole” on Earth, has experienced increasing N deposition rates in recent decades (Stocker et al., 2013). N deposition rates on the QTP range from 8.7 to 13.8 kg N ha^–^^1^ year^–^^1^ and are likely to increase in the coming decades (Lü and Tian, 2007). Alpine grasslands are the largest ecosystem on the QTP (Wang et al., 2012a; Zhao et al., 2017) and provide important life-supporting services for millions of people (Dong et al., 2010). These crucial plant communities are being degraded by numerous stressors, including climate change (Wen et al., 2010), and are likely to be especially sensitive to N deposition.

The objective of our study was to assess the response of plants in an alpine meadow of the QTP to increasing N deposition. We conducted a 3-year field experiment using four levels of N (0, 8, 40, and 72 kg N ha^–1^ yr^–1^) to simulate atmospheric N deposition. In this experiment, the nitrogen addition level of 8 kg N ha^–1^ year^–1^ was set according to the background value of atmospheric nitrogen deposition in this area (8.7–13.8 kg N ha^–1^ year^–1^) (Lü and Tian, 2007). Galloway et al. (2004) pointed that the N deposition rate on the Qinghai-Tibetan Plateau is predicted to increase to 40 kg N ha^–1^ year^–1^ by 2050, and this rate is estimated to be the N saturation threshold in alpine meadow (Zong et al., 2016). So in our experiment, the setting of 40 and 72 kg N ha^–1^ yr^–1^ was to simulate medium and high nitrogen deposition rate, respectively (Galloway et al., 2004; Bowman et al., 2012; Zong et al., 2016). Moderate nitrogen input can usually increase plant aboveground productivity (Xia and Wan, 2008). However, excessive N input can cause species loss through shifting plant communities to compositions that can tolerate high N levels (Bowman et al., 2008; Clark and Tilman, 2008). Additionally, excessive N input can substantially change soil nutrient balance and decrease soil pH (Phoenix et al., 2012), which may be the main reason that cause species loss (Stevens et al., 2010). Previous studies found that rhizomatous grasses are better adapted to high N levels (Bai et al., 2010). In addition, grasses species are in the upper part of meadow canopy and more competitive for light and soil nutrients (Hautier et al., 2009). Previous study found that grasses species such as *Poa pratensis* and *Stipa aliena* in alpine meadow had a more high N use efficiency with exogenous nitrogen input (Wang et al., 2012b). Based on the above studies, we hypothesized that nitrogen deposition would (1) decrease species richness, (2) increase the abundance and dominance of graminoids (mostly grasses), and (3) increase productivity, primarily through alterations in species composition.

## Materials and Methods

### Study Site

**FIGURE 1:**
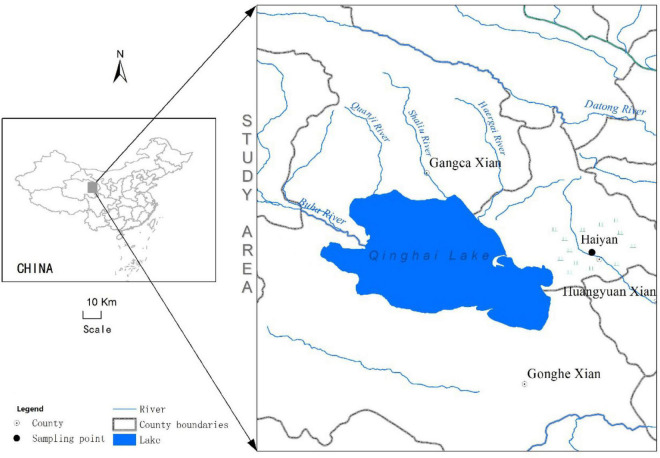
Location of the study site in the Qinghai-Tibetan Plateau of China.

**FIGURE 2:**
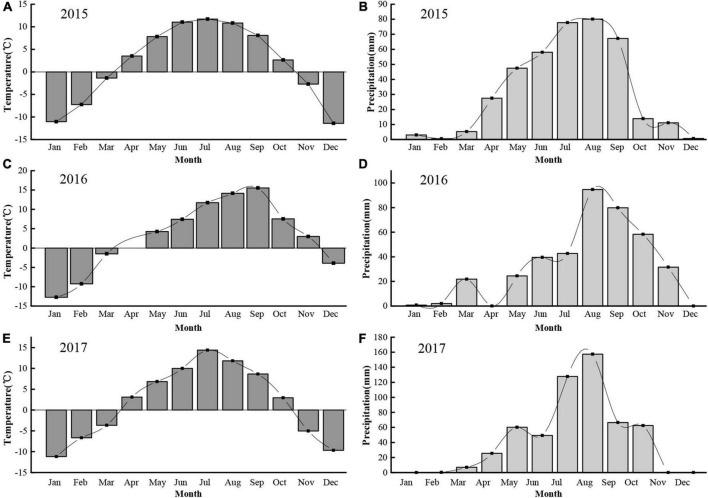
Monthly temperature (°C) and precipitation (mm) during each of 3 years at the study site. **(A,B)** Monthly temperature and precipitation in 2015, respectively; **(C,D)** Monthly temperature and precipitation in 2016, respectively; **(E,F)** Monthly temperature and precipitation in 2017, respectively.

We conducted our study in an alpine meadow near the town of Xihai, Haiyan County (36°56′N, 100°57′E, 3,100 m a.s.l.) in the Qinghai province of China (Figure 1). The soil at the study site is comprised primarily of clay. The study site had been fenced (1.2 m high) since 2012 to prevent entry by resident mammalian herbivores such as yak and sheep. The growing season in this region typically begins in early May and ends in late September. During the experimental years of 2015, 2016, and 2017, the annual rainfall totaled 392.8, 396.5 and 556.3 mm, respectively, while the mean annual temperature was 1.8, 3.3, and 1.8°C, respectively. The highest temperatures and precipitation generally occurred from April to October of each year (Figure 2).

### Experimental Design

We initiated the N addition treatments to simulate N deposition in 2014 on 12 plots, each measuring 2 by 5 m. All plots had similar topography, vegetation, and land-use history. Treatments comprised four N addition rates with three replicates for each rate: 0 kg N ha^–1^ yr^–1^ (control-CK), 8 kg N ha^–1^ yr^–1^ (Low-N), 40 kg N ha^–1^ yr^–1^ (Mid-N), and 72 kg N ha^–1^ yr^–1^ (High-N). We selected these N addition rates based on current and projected N deposition levels for the region (Lü and Tian, 2007). We applied nitrogen fertilizer (powder) to plots as ammonium nitrate (NH_4_NO_3_) in early May and July from 2014 to 2017 at dusk on a rainy day to avoid needing to water.

### Vegetation and Soil Sampling

We carried out vegetation surveys as well as biomass harvests and soil sampling in July and early-August of each year at peak vegetation growth for the region. Within each of the 12 plots, we identified each plant species in a randomly placed 1 by 1 m quadrat and recorded its abundance by point intercept method. In the same quadrats, another survey was carried out to visually estimate the percentage cover (visual estimation) of two functional plant groups: graminoids and non-graminoids. We used these data to calculate the graminoid dominance, the ratio of graminoid percentage cover to forb percentage cover. The graminoid dominance is a measure of functional composition. We also harvested the aboveground biomass of plants of the two functional groups in a 0.5 by 0.5 m sub-quadrat in each plot. Harvested plant samples oven-dried at 65°C (firstly oven-dried at 105°C for 3 h for de-enzyming) to constant weight. Lastly, we collected three soil cores in each plot using a 3.5 cm-diameter soil probe at a depth of 20 cm. Soil samples were mixed, air-dried at 70°C to constant weight, and sieved through a 0.15-mm mesh. We determined total nitrogen (TN) and carbon (TC) content of each composite soil sample using an elemental analyzer (EuroEA 3000, Pavia, Italy).

### Statistical Analysis

We performed all statistical analyses using SPSS (SPSS for Windows, version 22.0). We tested the effects of N addition on graminoids dominance (value of graminoids cover/non-graminoids cover), species richness, and productivity using one-way analysis of variance (ANOVA) with treatment means separated using the least significant differences (LSD) test at the *P* < 0.05 level. We used a two-way ANOVA to evaluate interactions between N addition and year. We used least-squares regression to assess relationships between graminoids dominance, species richness, and aboveground biomass. We evaluated associations between these community metrics, soil measurements, and climate factors with principal component analysis (PCA); Non-metric multidimensional scaling (NMDS) to estimate the differences in overall community composition across the treatments, and all these two analyses were carried out in R (version 4.1.2). To test the significance of the observed associations and understand their functional underpinnings that N addition and climatic factors affected plant productivity. The software package AMOS 22.0 was used to develop the SEM model and calculate related path coefficients, squared multiple correlations, direct and indirect effects and model fit. An insignificant χ^2^-statistic indicates that the SEM model provides a good fit. A qualified structural equation model (SEM) should match the criterion below (Jonsson and Wardle, 2010; Wei et al., 2013): (1) non-significant χ^2^ test, namely *P* > 0.05; (2) Comparative fit index, CFI > 0.95; (3) Root mean square error of approximation, RMSEA < 0.05.

## Results

### Effects of Nitrogen Deposition on Species and Functional Group Composition

**FIGURE 3:**
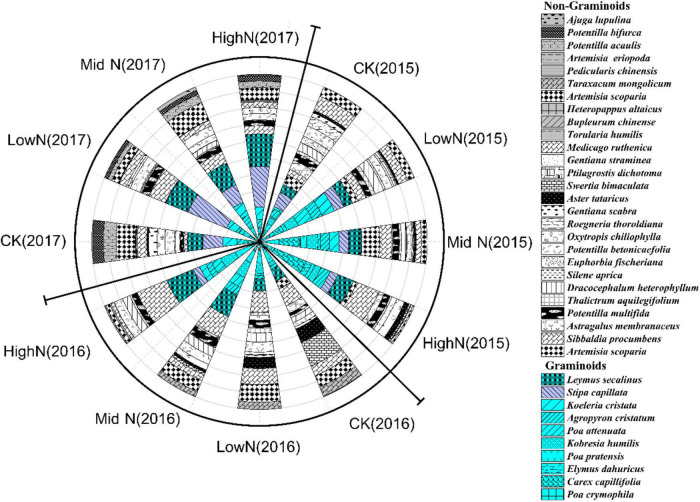
Effect of nitrogen addition rate on species composition based on abundance from 2015 to 2017. Species denoted in color are graminoids (mostly grasses).

**FIGURE 4:**
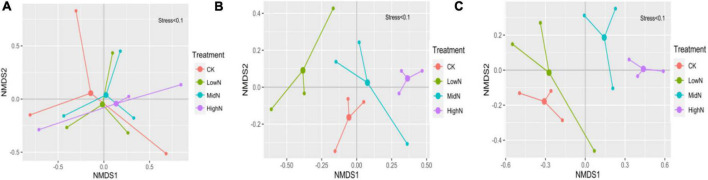
Non-metric multidimensional scaling (NMDS) plot of species composition from 2015 to 2017 under different treatments. **(A)** 2015; **(B)** 2016; **(C)** 2017. 0 kgN ha^–1^ yr^–1^ (CK), 8 kgN ha^–1^ yr^–1^ (LowN), 40 kgN ha^–1^ yr^–1^ (MidN), and 72 kg N ha^–1^ yr^–1^ (HighN).

**FIGURE 5:**
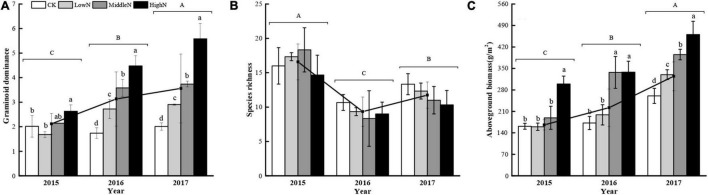
Effects of nitrogen addition rate on **(A)** graminoid ratio, **(B)** species richness, and **(C)** aboveground biomass during the 3-year study. Vertical bars indicate standard deviation of the mean. Lowercase letters indicate significant differences among nitrogen addition rates within years and uppercase letters indicate significant inter-annual differences (*P* < 0.05). Functional composition is expressed as the ratio of graminoid cover relative to the cover of non-graminoids (i.e., graminoid dominance). 0 kg N ha^–1^ yr^–1^ (CK), 8 kg N ha^–1^ yr^–1^ (LowN), 40 kg N ha^–1^ yr^–1^ (MidN), and 72 kg N ha^–1^ yr^–1^ (HighN).

The species composition of our alpine meadow plant community was markedly altered following the 3 years of N addition (Figures 3, 4). In plots subjected to the highest N addition rate, the abundance of grass species such as *Leymus secalinus*, *Agropyron cristatum*, *Poa crymophila*, and *Stipa purpurea* increased substantially during the experimental period while the abundance of forb species such as *Aster tataricus* and *Artemisia scoparia* decreased. There was a significant interaction between N treatment and year on the abundance of graminoids (mostly grasses) relative to non-graminoids (*F* = 12.72, *P* < 0.05, Table 1). In 2015, the first year of sampling after N addition, the abundance of graminoids relative to non-graminoids was greatest at the intermediate and highest N levels. In 2016, the relative abundance of graminoids was greatest at the highest N level. In 2017, following 3 years of N addition, the relative abundance of graminoids increased with increasing N addition rate. The graminoids ratio, the percent cover of graminoids relative to non-graminoids, increased significantly (*P* < 0.05) from 2015 to 2017 and this trend strengthened with increasing N addition. Species richness was not affected by N addition (Figure 5A) rate in our 3-year experiment but varied by year (*F* = 33.26, *P* < 0.05, Table 1). Species richness decreased significantly in 2016 and 2017 relative to 2015 (Figure 5B). Species richness was negatively associated with mean annual temperature (1.8°C in 2015, 3.3°C in 2016, and 1.8°C in 2017).

**TABLE 1:** Two-way ANOVA for the effects of year and N-addition rate on species richness, species composition, and total aboveground biomass.

Factors	Species richness		Species composition		Aboveground biomass	
	df	*F*	df	*F*	df	*F*
Year	2	33.26*	2	65.63*	2	85.95*
Nitrogen	3	1.401	3	89.97*	3	57.48*
Nitrogen × Year	6	0.921	6	12.72*	6	3.76*

*Significant according to a LSD test (P < 0.05).

### Effect of Nitrogen Deposition on Plant Aboveground Biomass

There was a significant interaction between N addition level and year (*F* = 3.76, *P* < 0.05, Table 1) on aboveground biomass. In 2015, after 1 year of N addition, the aboveground biomass of plants subjected to the highest N addition rate was greater than all other treatments (Figure 5C). In 2016, plants in the two highest N addition treatments had greater biomass than the control and lowest N addition treatment. In 2017, after 3 years of treatments, aboveground biomass increased with each increase in N addition rate. The total aboveground biomass across N addition levels increased significantly between 2015 and 2017 (Figure 5C and Table 1).

### Relationships Between Species Richness, Functional Group Composition, and Aboveground Biomass

**FIGURE 6:**
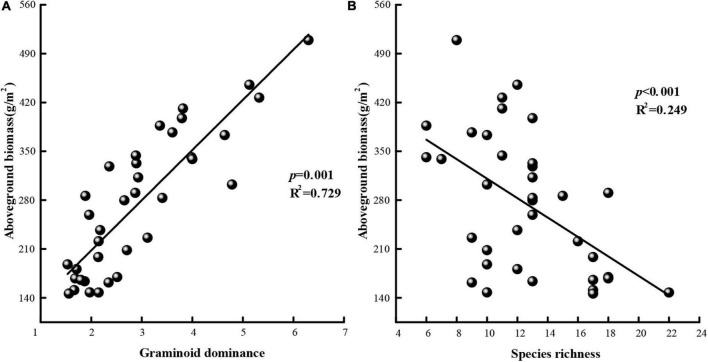
Aboveground biomass in terms of **(A)** graminoid dominance and **(B)** species richness. Fitted lines are based on least-squares regression. Graminoid dominance is expressed as the ratio of graminoid cover relative to the cover of non-graminoids.

Using data for all 3 years and least-squares regression analysis, we evaluated the relationship between aboveground biomass and functional group composition (i.e., graminoid dominance) (Figure 6A) and the relationship between aboveground biomass and species richness (Figure 6B). Increasing graminoid dominance was positively correlated with total aboveground biomass (*R*^2^ = 0.729, *P* = 0.001) while species richness was negatively correlated with the total aboveground biomass (*R*^2^ = 0.249, *P* < 0.001).

### Direct and Indirect Drivers That Affect Plant Productivity

**FIGURE 7:**
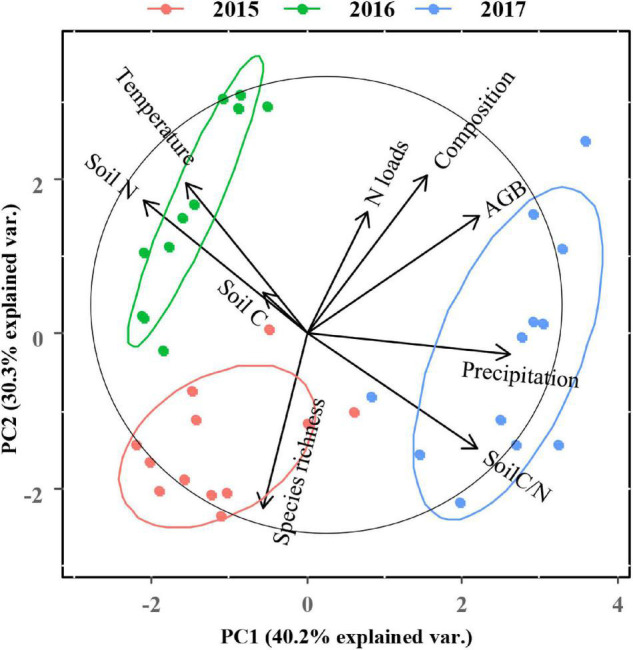
Principal component analysis (PCA) for all plant community parameters, soil properties, temperature, and precipitation across different N addition rates during the 3-year field study. The length of a variable vector in the representation space is indicative of the contribution level of the variable. Composition is expressed as graminoid cover/non-graminoids (i.e., graminoid dominance). AGB, aboveground biomass.

**FIGURE 8:**
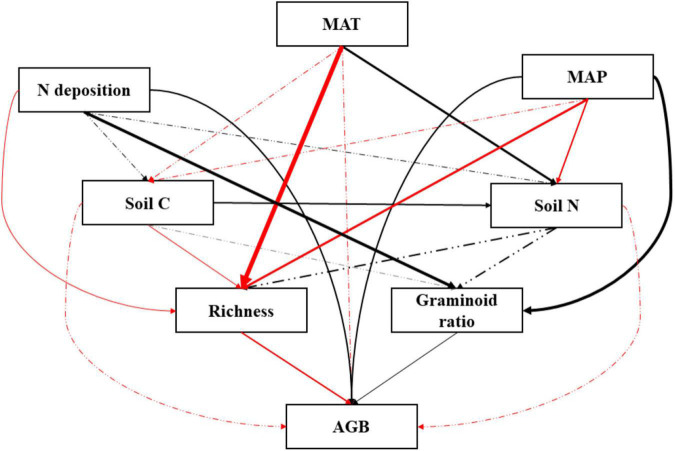
Structural equation model (SEM) testing the relationships between community, soil, and climatic variables. Significant impacts are shown by solid lines whereas non-significant impacts are shown by dashed-dotted lines. Arrow width is proportional to the strength of the relationship. Positive impacts are shown in black and negative impacts are shown in red. MAT, mean annual temperature; MAP, mean annual precipitation; Soil C, soil carbon content; Soil N, soil nitrogen content; AGB, aboveground biomass. χ^2^ = 3.066, *P* = 0.382, GFI = 0.979, NFI = 0.989, RMSEA = 0.025.

A PCA analysis of plant community performance, soil properties, and climatic factors revealed that PC1 and PC2 explained 70.5% of the variance (Figure 7). Data were clearly separated by year. We found that soil N and soil C/N were the two variables contributing most to the patterns observed. The graminoid dominance and the total aboveground biomass were positively related to N addition rates and mean annual precipitation. We also developed an SEM model (Figure 8, χ^2^ = 3.066, *P* = 0.382, GFI = 0.979, NFI = 0.989, RMSEA = 0.025) showing that N addition and climate can influence aboveground community biomass indirectly as well as directly. Nitrogen addition increased aboveground biomass largely by increasing the graminoid dominance. Precipitation influenced aboveground biomass through its effects on both the graminoid dominance and species richness. Lastly, higher mean annual temperatures affected aboveground biomass by decreasing species richness.

## Discussion

### Inter-Annual Variability in Species Richness

Species richness is a fundamental component of biodiversity. Despite numerous studies, the relationship between productivity and the species richness of a community remains unclear (Mittelbach et al., 2001; Adler et al., 2011). This relationship has sometimes been characterized as a hump-back response in temperate vegetation (Oba et al., 2001). The hump-back model has received some support on the Qinghai-Tibetan Plateau, although productivity-diversity relationships in this region show considerable variation associated with variation in precipitation and species composition (Wu et al., 2014). The downhill slope of the hump-back model may be consistent with previous reports of declines in species richness following N deposition (Bai et al., 2010; Damgaard et al., 2011; Fang et al., 2012). In our study, the impact of N deposition on species richness was not significant in any of the 3 years, although we did observe a decreasing trend with increasing N in 2016 and 2017. We also observed that species richness was negatively correlated with aboveground biomass across the entire dataset.

We found that more variation in species richness was explained by year than by N deposition rates. Average species richness across treatments declined from 17 species in 2015 to 9 species in 2016, and then increased back to 12 species in 2017. These fluctuations in species richness appeared to be inversely associated with mean annual temperatures. Elevated ambient temperatures have been reported to reduce the number of plant species in this region of China (Klein et al., 2004). Additional research and longer-term studies should evaluate this negative relationship between temperature and species richness, which may have significant implications for the stability of sensitive alpine grassland communities in the context of climate change.

### Nitrogen Availability and Climate Jointly Influence Productivity

Nitrogen is a limiting soil nutrient in many terrestrial ecosystems (LeBauer and Treseder, 2008). N deposition can improve soil N availability and thereby promote plant productivity (Bai et al., 2010; Liu et al., 2011; Zhang et al., 2015). In the first year of our study, we found that aboveground biomass increased significantly under the high N deposition rate but did not change substantially under the low and intermediate N deposition rates. In the next 2 years, aboveground biomass increased with the rate of N deposition. These results suggest that N may be a critical limiting nutrient for plant productivity in this region, yet there exists hysteresis effect of N accumulation. In this study, soil N and soil C/N were the two variables contributing most to the patterns observed. The graminoid dominance and the total aboveground biomass were positively related to N addition rates and mean annual precipitation. This suggests that soil nutrients which are classically considered to be variable and in this system may be most strongly controlled mechanistically by amount of rainfall, and both soil C and N which classically only change with long-term or very disruptive manipulations ecosystem change are what is driving separation in multivariate space among years. Our findings are consistent with previous studies showing N limitation in grassland ecosystems (Bassin et al., 2007; Dai et al., 2019). Alpine grasslands are usually N-limited, therefore N deposition can release such nutrient restriction to some degree. Our results substantiated previous conclusions that N deposition can enhance alpine grassland productivity (Liu et al., 2011; Zhang et al., 2015), however, hysteresis effect existed as N loads and time length of N deposition increase. Also, climatic factors such as precipitation can obviously interact with N deposition to influence productivity.

We also observed interannual differences in aboveground plant productivity. These differences are consistent with the idea that N availability interacts with climatic variables, such as temperature and precipitation, to affect plant productivity in grassland ecosystems (Harpole et al., 2007; Sala et al., 2012). Water availability is a particularly important influence on plant responses to N (Hooper and Johnson, 1999; Bai et al., 2008), primarily because increasing productivity increases transpiration, potentially exacerbating water shortages (Harpole et al., 2007). In our study, the effects of N deposition on plant productivity were greatest in the year with the highest precipitation (2017). Our SEM modeling analysis also showed that precipitation may intensify the effects of N on plant productivity. Mean annual precipitation increased from 2015 to 2017, and the mean annual precipitation was highest among 3 years, also the plant community AGB significantly increased over years, suggesting that a higher water availability induced by high precipitation will also favor the grassland productivity in alpine ecosystem. In addition, the high precipitation period was mainly during the growing season (July∼August), indicating that summer precipitation variability plays an important role in determine the increase of grassland productivity. Soil water content is related to temperature and precipitation, higher temperature can cause water evaporation. During the growing season, the temperature increased over years, and precipitation showed the same variation trend, suggesting that the alpine regions is experiencing a warming and more humid environment change. Therefore, soil water content might increase if the increase of soil water availability induced by precipitation can override water loss of evaporation under warmer climate. Our previous study has found that warming could cause water loss in alpine meadow (Shen et al., 2020). In cold alpine regions, temperature and precipitation are usually the limiting factors for plants, though warmer could accelerate water evaporation, some studies reckoned that a higher temperature may favor plant growth by releasing cold-limitation in alpine regions (Ganjurjav et al., 2016). However, relation between temperature and soil nutrients is not clear (Kladivko and Keeney, 1987; Sardans et al., 2008; Wang et al., 2016). Higher temperature can cause more soil water evaporation, such water loss is detrimental for plant nutrient uptake as the acquisition of nutrients by plants depends mostly on soil water availability (Querejeta et al., 2021). Yet, at least in our study such negative effects are not obvious than the positive effects from the perspective of community productivity level. Community productivity increases can likely occur in the warmer and wetter alpine regions in the future (Winkler et al., 2016).

### Shifts in Community Composition Help Explain Trends in Productivity

Previous nutrient addition studies have reported increases in the aboveground biomass and cover of graminoids with increasing N and concomitant decreases in the dominance of non-graminoids (Stevens et al., 2006; Song et al., 2011; Fang et al., 2012). In agreement with such studies, we found that N deposition increased the abundance and cover of graminoids over non-graminoids. Our data also indicated that N deposition could shift plant species composition in favor of graminoid species within a 3 years period.

We found that the graminoid dominance was positively correlated with community productivity. This finding is consistent with previous research showing that increases in productivity following N deposition were largely due to increases in the prevalence and aboveground growth of grasses (Xu et al., 2015; Fu and Shen, 2016; You et al., 2017). In our study site, grasses grew much taller than non-graminoids and likely outcompeted forb species for light after N deposition. Grasses have also been shown to have higher soil nutrient-use efficiencies, especially for soil N (Huang et al., 2008). Lastly, N deposition can lead to soil acidification and accumulation of Mn^2+^, which reduces photosynthetic rates more in non-graminoids than grasses (Tian et al., 2016). The differential effects of N on grasses and non-graminoids suggest that N deposition is likely to have significant effects on community function in the long-term (Shaver et al., 2001).

## Conclusion

Simulated N deposition led to large differences in plant community performance in an alpine meadow of the Qinghai-Tibetan Plateau over 3 years. Nitrogen deposition significantly increased the total aboveground biomass of the plant community and shifted community composition in favor of graminoids. These results suggest that continued N deposition in alpine meadow could significantly alter plant communities diversity, function, and stability. Increased productivity of these generally N-limited habitats could support greater densities of wild or domesticated grazing animals, but the long-term potential effects of N on these sensitive ecosystems are largely unknown. The effects of temperature and precipitation on species richness and plant community responses to N also require further research. Our 3-year field study demonstrates that N deposition and climate variability can have extensive impacts on alpine grassland plant communities, even over short periods. These changes are likely to be magnified if N deposition persists over longer time periods or interacts with the effects of climate change.

## Data Availability Statement

The raw data supporting the conclusions of this article will be made available by the authors, without undue reservation.

## Author Contributions

SD designed the study. HS analyzed the data and contributed to writing. SD and AD helped to revising the manuscript. HS, JX, YZ, and LW carried out the experiment. All authors contributed to the article and approved the submitted version.

## Conflict of Interest

The authors declare that the research was conducted in the absence of any commercial or financial relationships that could be construed as a potential conflict of interest.

## Publisher’s Note

All claims expressed in this article are solely those of the authors and do not necessarily represent those of their affiliated organizations, or those of the publisher, the editors and the reviewers. Any product that may be evaluated in this article, or claim that may be made by its manufacturer, is not guaranteed or endorsed by the publisher.

## References

[B1] Adler P. B. Seabloom E. W. Borer E. T. Hillebrand H. Hautier Y. Hector A. (2011). Productivity is a poor predictor of plant species richness. *Science* 333 1750–1753. 21940895 10.1126/science.1204498

[B2] Bai Y. Wu J. Clark C. M. Naeem S. Pan Q. Huang J. (2010). Tradeoffs and thresholds in the effects of nitrogen addition on biodiversity and ecosystem functioning: evidence from inner Mongolia Grasslands. *Glob. Change Biol.* 16 358–372.

[B3] Bai Y. Wu J. Xing Q. Pan Q. Huang J. Yang D. (2008). Primary production and rain use efficiency across a precipitation gradient on the Mongolia plateau. *Ecology* 89 2140–2153. 10.1890/07-0992.1 18724724

[B4] Bassin S. Volk M. Suter M. Buchmann N. Fuhrer J. (2007). Nitrogen deposition but not ozone affects productivity and community composition of subalpine grassland after 3 years of treatment. *New Phytol.* 175 523–534. 10.1111/j.1469-8137.2007.02140.x 17635227

[B5] Bobbink R. Hicks K. Galloway J. Spranger T. Alkemade R. Ashmore M. (2010). Global assessment of nitrogen deposition effects on terrestrial plant diversity: a synthesis. *Ecol. Appl.* 20 30–59. 10.1890/08-1140.1 20349829

[B6] Bowman W. D. Cleveland C. C. Halada Å Hreško J. Baron J. S. (2008). Negative impact of nitrogen deposition on soil buffering capacity. *Nat. Geosci.* 1 767–770. 10.1038/ngeo339

[B7] Bowman W. D. Murgel J. Blett T. Porter E. (2012). Nitrogen critical loads for alpine vegetation and soils in Rocky Mountain National Park. *J. Environ. Manag.* 103 165–171. 10.1016/j.jenvman.2012.03.002 22516810

[B8] Clark C. M. Tilman D. (2008). Loss of plant species after chronic low-level nitrogen deposition to prairie grasslands. *Nature* 451:712. 10.1038/nature06503 18256670

[B9] Dai L. Ke X. Du Y. Zhang F. Li Y. Li Q. (2019). Nitrogen controls the net primary production of an alpine Kobresia meadow in the northern Qinghai−Tibet Plateau. *Ecol.Evol.* 9 8865–8875. 10.1002/ece3.5442 31410286 PMC6686337

[B10] Damgaard C. Jensen L. Frohn L. M. Borchsenius F. Nielsen K. E. Ejrnæs R. (2011). The effect of nitrogen deposition on the species richness of acid grasslands in Denmark: a comparison with a study performed on a European scale. *Environ. Pollut.* 159 1778–1782. 10.1016/j.envpol.2011.04.003 21530033

[B11] Dong S. Wen L. Zhu L. Li X. (2010). Implication of coupled natural and human systems in sustainable rangeland ecosystem management in HKH region. *Front. Earth Sci. PRC* 4 42–50. 10.1007/s11707-010-0010-z

[B12] Fang Y. Xun F. Bai W. Zhang W. Li L. (2012). Long-term nitrogen addition leads to loss of species richness due to litter accumulation and soil acidification in a temperate steppe. *PLoS One* 7:e47369. 10.1371/journal.pone.0047369 23077603 PMC3470592

[B13] Fu G. Shen Z. X. (2016). Response of alpine plants to nitrogen addition on the Tibetan Plateau: A meta-analysis. *J. Plant Growth Regul.* 35 974–979. 10.1007/s00344-016-9595-0

[B14] Galloway J. N. Dentener F. J. Capone D. G. Boyer E. W. Howarth R. W. Seitzinger S. P. (2004). Nitrogen cycles: past, present, and future. *Biogeochemistry* 70 153–226.

[B15] Galloway J. N. Dentener F. J. Marmer E. Cai Z. Abrol Y. P. Dadhwal V. K. (2008a). The environmental reach of Asia. *Annu. Rev. Env. Resour.* 33 461–481.

[B16] Galloway J. N. Townsend A. R. Erisman J. W. Bekunda M. Cai Z. Freney J. R. (2008b). Transformation of the nitrogen cycle: recent trends, questions, and potential solutions. *Science* 320 889–892. 10.1126/science.1136674 18487183

[B17] Ganjurjav H. Gao Q. Gornish E. S. Schwartz M. W. Liang Y. Cao X. (2016). Differential response of alpine steppe and alpine meadow to climate warming in the central Qinghai–Tibetan Plateau. *Agr. Forest Meteorol.* 223 233–240. 10.1016/j.agrformet.2016.03.017

[B18] Gruber N. Galloway J. N. (2008). An earth-system perspective of the global nitrogen cycle. *Nature* 451 293–296. 10.1038/nature06592 18202647

[B19] Harpole W. S. Tilman D. (2007). Grassland species loss resulting from reduced niche dimension. *Nature* 446 791–793. 10.1038/nature05684 17384633

[B20] Harpole W. S. Potts D. L. Suding K. N. (2007). Ecosystem responses to water and nitrogen amendment in a California grassland. *Global Change Biol.* 13 2341–2348. 10.1111/j.1365-2486.2007.01447.x

[B21] Hautier Y. Niklaus P. A. Hector A. (2009). Competition for light causes plant biodiversity loss after eutrophication. *Science* 324 636–638. 10.1126/science.1169640 19407202

[B22] Hector A. Schmid B. Beierkuhnlein C. (1999). Plant diversity and productivity experiments in European grasslands. *Science* 286 1123–1127. 10.1126/science.286.5442.1123 10550043

[B23] Hooper D. U. Johnson L. (1999). Nitrogen limitation in dryland ecosystems: responses to geographical and temporal variation in precipitation. *Biogeochemistry* 46 247–293.

[B24] Hooper D. U. Vitousek P. M. (1997). The effects of plant composition and diversity on ecosystem processes. *Science* 277 1302–1305. 10.1126/science.277.5330.1302

[B25] Huang J. Y. Zhu X. G. Yuan Z. Y. Song S. H. Li X. Li L. H. (2008). Changes in nitrogen resorption traits of six temperate grassland species along a multi-level N addition gradient. *Plant Soil* 306 149–158.

[B26] Hui D. Jackson R. B. (2006). Geographical and interannual variability in biomass partitioning in grassland ecosystems: a synthesis of field data. *New Phytol.* 169 85–93. 10.1111/j.1469-8137.2005.01569.x 16390421

[B27] Isbell F. Tilman D. Polasky S. Binder S. Hawthorne P. (2013). Low biodiversity state persists two decades after cessation of nutrient enrichment. *Ecol. Lett.* 16 454–460. 10.1111/ele.12066 23301631

[B28] Jonsson M. Wardle D. A. (2010). Structural equation modelling reveals plant-community drivers of carbon storage in boreal forest ecosystems. *Biol. Lett.* 6 116–119. 10.1098/rsbl.2009.0613 19755530 PMC2817262

[B29] Kladivko E. J. Keeney D. R. (1987). Soil nitrogen mineralization as affected by water and temperature interactions. *Biol. Fert. Soils* 5 248–252.

[B30] Klein J. A. Harte J. Zhao X. Q. (2004). Experimental warming causes large and rapid species loss, dampened by simulated grazing, on the Tibetan Plateau. *Ecol. Lett.* 7 1170–1179.

[B31] LeBauer D. S. Treseder K. K. (2008). Nitrogen limitation of net primary productivity in terrestrial ecosystems is globally distributed. *Ecology* 89 371–379. 10.1890/06-2057.1 18409427

[B32] Liu X. Duan L. Mo J. Du E. Shen J. Lu X. (2011). Nitrogen deposition and its ecological impact in China: an overview. *Environ. Pollut.* 159 2251–2264. 10.1016/j.envpol.2010.08.002 20828899

[B33] Lü C. Tian H. (2007). Spatial and temporal patterns of nitrogen deposition in China: synthesis of observational data. *J. Geophys. Res. Atmos.* 112:D22.

[B34] Marini L. Scotton M. Klimek S. Isselstein J. Pecile A. (2007). Effects of local factors on plant species richness and composition of Alpine meadows. *Agric. Ecosyst. Environ.* 119 281–288. 10.1038/srep18161 26670681 PMC4680968

[B35] Mittelbach G. G. Steiner C. F. Scheiner S. M. Gross K. L. Reynolds H. L. Waide R. B. (2001). What is the observed relationship between species richness and productivity? *Ecology* 82 2381–2396.

[B36] Mountford J. O. Lakhani K. H. Kirkham F. W. (1993). Experimental assessment of the effects of nitrogen addition under hay-cutting and aftermath grazing on the vegetation of meadows on a Somerset peat moor. *J. Appl. Ecol.* 1993 321–332. 10.2307/2404634

[B37] Myklestad Å (2004). Soil, site and management components of variation in species composition of agricultural grasslands in western Norway. *Grass Forage Sci.* 59 136–143. 10.1111/j.1365-2494.2004.00413.x

[B38] Oba G. Vetaas O. R. Stenseth N. C. (2001). Relationships between biomass and plant species richness in arid−zone grazing lands. *J. Appl. Ecol.* 38 836–845. 10.1046/j.1365-2664.2001.00638.x

[B39] Phoenix G. K. Emmett B. A. Britton A. J. Caporn S. J. Dise N. B. Helliwell R. (2012). Impacts of atmospheric nitrogen deposition: responses of multiple plant and soil parameters across contrasting ecosystems in long−term field experiments. *Glob. Change Biol.* 18 1197–1215. 10.1111/j.1365-2486.2011.02590.x

[B40] Phoenix G. K. Hicks W. K. Cinderby S. Kuylenstierna J. C. Stock W. D. Dentener F. J. (2006). Atmospheric nitrogen deposition in world biodiversity hotspots: the need for a greater global perspective in assessing N deposition impacts. *Glob. Change Biol.* 12 470–476.

[B41] Pierik M. Van Ruijven J. Bezemer T. M. Geerts R. H. Berendse F. (2011). Recovery of plant species richness during long−term fertilization of a species−rich grassland. *Ecology* 92 1393–1398. 10.1890/10-0210.1 21870612

[B42] Querejeta J. I. Ren W. Prieto I. (2021). Vertical decoupling of soil nutrients and water under climate warming reduces plant cumulative nutrient uptake, water−use efficiency and productivity. *New Phytol.* 230 1378–1393. 10.1111/nph.17258 33550582

[B43] Sala O. E. Gherardi L. A. Reichmann L. Jobbagy E. Peters D. (2012). Legacies of precipitation fluctuations on primary production: theory and data synthesis. *Philos. T. R. Soc. B* 367 3135–3144. 10.1098/rstb.2011.0347 23045711 PMC3479688

[B44] Sardans J. Peñuelas J. Prieto P. Estiarte M. (2008). Changes in Ca, Fe, Mg, Mo, Na, and S content in a Mediterranean shrubland under warming and drought. *J. Geophys. Res. Biogeo.* 113:795.

[B45] Shaver G. R. Bret-Harte M. S. Jones M. H. Johnstone J. Gough L. Laundre J. (2001). Species composition interacts with fertilizer to control long−term change in tundra productivity. *Ecology* 82 3163–3181. 10.1890/15-1160.1 27459784

[B46] Shen H. Dong S. Li S. Wang W. Xiao J. Yang M. (2020). Effects of warming and N deposition on the physiological performances of Leymus secalinus in alpine meadow of Qinghai-Tibetan Plateau. *Front. Plant Sci.* 10:1804. 10.3389/fpls.2019.01804 32153598 PMC7047333

[B47] Shen H. Dong S. Li S. Xiao J. Han Y. Yang M. (2019). Effects of simulated N deposition on photosynthesis and productivity of key plants from different functional groups of alpine meadow on Qinghai-Tibetan plateau. *Environ. Pollut.* 251 731–737. 10.1016/j.envpol.2019.05.045 31112927

[B48] Song L. Bao X. Liu X. Zhang Y. Christie P. Fangmeier A. (2011). Nitrogen enrichment enhances the dominance of grasses over forbs in a temperate steppe ecosystem. *Biogeosciences* 8 2341–2350. 10.5194/bg-8-2341-2011

[B49] Stevens C. J. Dise N. B. Gowing D. J. Mountford J. O. (2006). Loss of forb diversity in relation to nitrogen deposition in the UK: regional trends and potential controls. *Glob. Change Biol.* 12 1823–1833. 10.1111/j.1365-2486.2006.01217.x

[B50] Stevens C. J. Dise N. B. Mountford J. O. Gowing D. J. (2004). Impact of nitrogen deposition on the species richness of grasslands. *Science* 303 1876–1879. 10.1126/science.1094678 15031507

[B51] Stevens C. J. Duprè C. Dorland E. Gaudnik C. Gowing D. J. Bleeker A. (2010). Nitrogen deposition threatens species richness of grasslands across Europe. *Environ. Pollut.* 158 2940–2945. 10.1016/j.envpol.2010.06.006 20598409

[B52] Stevens C. J. Lind E. M. Hautier Y. Harpole W. S. Borer E. T. Hobbie S. (2015). Anthropogenic nitrogen deposition predicts local grassland primary production worldwide. *Ecology* 96 1459–1465. 10.1890/14-1902.1

[B53] Stocker T. F. Qin D. Plattner G.-K. Tignor M. Allen S. K. Boschung J. (2013). *Climate Change 2013: The Physical Science Basis. Contribution of Working Group I to the 5th Assessment Report of the Intergovernmental Panel on Climate Change.* Cambridge: Cambridge University Press, 1535.

[B54] Tian Q. Liu N. Bai W. Li L. Chen J. Reich P. B. (2016). A novel soil manganese mechanism drives plant species loss with increased nitrogen deposition in a temperate steppe. *Ecology* 97 65–74. 10.1890/15-0917.1 27008776

[B55] Tilman D. Wedin D. Knops J. (1996). Productivity and sustainability influenced by biodiversity in grassland ecosystems. *Nature* 379 718–720. 10.1038/379718a0

[B56] Vitousek P. M. Aber J. D. Howarth R. W. Likens G. E. Matson P. A. Schindler D. W. (1997). Human alteration of the global nitrogen cycle: sources and consequences. *Ecol. Appl.* 7 737–750. 10.2307/2269431

[B57] Vitousek P. M. Howarth R. W. (1991). Nitrogen limitation on land and in the sea: how can it occur? *Biogeochemistry* 13 87–115.

[B58] Wang G. X. Liu G. S. Li C. J. (2012a). Effects of changes in alpine grassland vegetation cover on hillslope hydrological processes in a permafrost watershed. *J. Hydrol.* 444–445 22–33. 10.1016/j.jhydrol.2012.03.033

[B59] Wang J. Zhang X. Li L. Cheng K. Zheng J. Zheng J. (2016). Changes in micronutrient availability and plant uptake under simulated climate change in winter wheat field. *J. Soil. Sediment.* 16 2666–2675.

[B60] Wang W. Ma Y. Xu J. Wang H. Zhu J. Zhou H. (2012b). The uptake diversity of soil nitrogen nutrients by main plant species in Kobresia humilis alpine meadow on the Qinghai-Tibet Plateau. *Sci. China Earth Sci.* 55 1688–1695. 10.1007/s11430-012-4461-9

[B61] Wei C. Yu Q. Bai E. Lü X. Li Q. I. Xia J. (2013). Nitrogen deposition weakens plant–microbe interactions in grassland ecosystems. *Glob. Change Biol.* 19 3688–3697. 10.1111/gcb.12348 23925948

[B62] Wen L. Dong S. K. Zhu L. Li X. Y. Shi J. J. Wang Y. L. (2010). The construction of grassland degradation index for alpine meadow in Qinghai-Tibetan Plateau. *Proc. Environ. Sci.* 2 1966–1969.

[B63] Winkler D. E. Chapin K. J. Kueppers L. M. (2016). Soil moisture mediates alpine life form and community productivity responses to warming. *Ecology* 97 1553–1563. 27859221 10.1890/15-1197.1

[B64] Wu J. Shen Z. Zhang X. (2014). Precipitation and species composition primarily determine the diversity–productivity relationship of alpine grasslands on the Northern Tibetan Plateau. *Alpine Bot.* 124 13–25.

[B65] Xia J. Wan S. (2008). Global response patterns of terrestrial plant species to nitrogen addition. *New Phytol.* 179 428–439. 10.1111/j.1469-8137.2008.02488.x 19086179

[B66] Xu D. Gao X. Gao T. Mou J. Li J. Bu H. (2018). Interactive effects of nitrogen and silicon addition on growth of five common plant species and structure of plant community in alpine meadow. *Catena* 169 80–89.

[B67] Xu X. Liu H. Song Z. Wang W. Hu G. Qi Z. (2015). Response of aboveground biomass and diversity to nitrogen addition along a degradation gradient in the Inner Mongolian steppe, China. *Sci. Rep.* 5:10284. 10.1038/srep10284 26194184 PMC4508527

[B68] You C. Wu F. Gan Y. Yang W. Hu Z. Xu Z. (2017). Grass and forbs respond differently to nitrogen addition: a meta-analysis of global grassland ecosystems. *Sci. Rep.* 7:1563. 10.1038/s41598-017-01728-x 28484219 PMC5431500

[B69] Zhang Y. Feng J. Isbell F. Lü X. Han X. (2015). Productivity depends more on the rate than the frequency of N addition in a temperate grassland. *Sci. Rep.* 5:12558. 10.1038/srep12558 26218675 PMC4517389

[B70] Zhao Z. Dong S. Jiang X. Liu S. Ji H. Li Y. (2017). Effects of warming and nitrogen deposition on CH_4_, CO_2_ and N_2_O emissions in alpine grassland ecosystems of the Qinghai-Tibetan Plateau. *Sci. Total Environ.* 592 565–572. 10.1016/j.scitotenv.2017.03.082 28318700

[B71] Zong N. Shi P. Song M. Zhang X. Jiang J. Chai X. (2016). Nitrogen critical loads for an alpine meadow ecosystem on the Tibetan Plateau. *Environ. Manage.* 57 531–542. 10.1007/s00267-015-0626-6 26475686

